# Dynamics of aggressive manifestations in eating disorders

**DOI:** 10.1192/j.eurpsy.2021.949

**Published:** 2021-08-13

**Authors:** I. Belokrylov, A. Bryukhin, T. Lineva, E. Okonishnikova

**Affiliations:** 1 Department Of Psychiatry And Medical Psychologi, Peoples Friendship University of Russia (RUDN University), Moscow, Russian Federation; 2 Department Of Psychiatry And Medical Psychology, Peoples Friendship University of Russia (RUDN University), Moscow, Russian Federation; 3 Department Of Psychiatry And Medical Psychology, RUDN University Moscow., Moscow, Russian Federation

## Abstract

**Introduction:**

Anorexia nervosa and bulimia nervosa are often accompanied by aggressive manifestations that undergo typical dynamics at different stages of the disease. The presence of aggressive phenomena in eating disorders can cause severe maladaptation of patients, cause difficulties in diagnosis, establishing compliance, and prevent the normalization of family relations.

**Objectives:**

To study the varieties of aggressive manifestations and their changes in the treatment of anorexia nervosa and bulimia.

**Methods:**

Psychopathological, anamnestic, psychological.

**Results:**

The most pronounced aggressive symptoms in typical anorexia nervosa are verbal and physical aggression against relatives and close people; feeding younger siblings, parents; threats and suppression of the opinion of relatives in relation to patients. The above aggressive statements and actions occur at the stage of correction and in the initial period of the stage of exhaustion. With deep exhaustion (pronounced cachexia) and in the process of food rehabilitation, aggressive behavior is significantly reduced. In the future, there is criticism of their own aggressive symptoms. In bulimia nervosa, only verbal aggression toward loved ones is noted, especially when they interfere with purifying behavior and massive compulsive overeating. The degree of aggression in bulimia nervosa is significantly less.

**Conclusions:**

Aggressive manifestations in eating disorders depend on the stage of the disease, the degree of exhaustion and undergo reverse development in the course of therapy. Aggressive phenomena in eating disorders have a significant impact on the clinic, dynamics, outcomes of diseases and the effectiveness of treatment tactics.

**Conflict of interest:**

No significant relationships.

